# Prenatal Ethanol Exposure and Whisker Clipping Disrupt Ultrasonic Vocalizations and Play Behavior in Adolescent Rats

**DOI:** 10.3390/brainsci6040043

**Published:** 2016-09-28

**Authors:** Jaylyn Waddell, Tianqi Yang, Eric Ho, Kristen A. Wellmann, Sandra M. Mooney

**Affiliations:** Department of Pediatrics, University of Maryland School of Medicine, Baltimore, MD 21201, USA; jwaddell@som.umaryland.edu (J.W.); tansygarvey@gmail.com (T.Y.); eho@peds.umaryland.edu (E.H.); k.wellmann@gmail.com (K.A.W.)

**Keywords:** fetal alcohol spectrum disorder, rat, sensory impoverishment, social behavior, USV

## Abstract

Prenatal ethanol exposure can result in social deficits in humans and animals, including altered social interaction and poor communication. Rats exposed to ethanol prenatally show reduced play fighting, and a combination of prenatal ethanol exposure and neonatal whisker clipping further reduces play fighting compared with ethanol exposure alone. In this study, we explored whether expression of hedonic ultrasonic vocalizations (USVs) correlated with the number of playful attacks by ethanol-exposed rats, rats subjected to postnatal sensory deprivation by whisker clipping or both compared to control animals. In normally developing rats, hedonic USVs precede such interactions and correlate with the number of play interactions exhibited in dyads. Pregnant Long-Evans rats were fed an ethanol-containing liquid diet or a control diet. After birth, male and female pups from each litter were randomly assigned to the whisker-clipped or non-whisker-clipped condition. Animals underwent a social interaction test with a normally developing play partner during early or late-adolescence. USVs were recorded during play. Prenatal ethanol exposure reduced both play and hedonic USVs in early adolescence compared to control rats and persistently reduced social play. Interestingly, ethanol exposure, whisker clipping and the combination abolished the significant correlation between hedonic USVs and social play detected in control rats in early adolescence. This relationship remained disrupted in late adolescence only in rats subjected to both prenatal ethanol and whisker clipping. Thus, both insults more persistently disrupted the relationship between social communication and social play.

## 1. Introduction

Ultrasonic vocalizations (USVs) are a critical aspect of social interaction in rats. Rodent USVs can be classified into two main categories in adolescence and adulthood: 50 kHz “hedonic” calls and 22 kHz “alarm” calls (e.g., [1]). The 50 kHz USVs are often associated with appetitive and rewarding contexts such as elicitation of social play and social approach, sex, food, and cocaine administration [2,3,4]. Alarm calls are typically elicited by distressing or alarming cues including painful or startling stimuli, predator indicators, or conspecific aggression [5,6,7,8].

The majority of 50 kHz USVs emitted during social interactions occur just prior to a social play interaction in which one rat playfully attacks another rat [9]. Rats selectively bred to express fewer USVs engage in fewer playful attacks and spend less time in social contact with a conspecific than those selectively bred to emit a high rate of USVs [10,11]. This pattern was evident in females in initial play interactions, and developed over days in male rats [10]. The 50 kHz USVs may serve to signal the playfulness and reciprocity of rough and tumble play, and/or avoid aggressive behavior or unwanted interaction (discussed in [9]). Because rough and tumble play can become aggressive, it is likely that hedonic USVs disambiguate the similar behaviors inherent to both play and aggression [9,12,13]. Playful interactions require rapid integration of input from multiple sensory domains, ranging from tactile to olfactory in rodents [14]. Thus, distinct USVs facilitate accurate interpretation of these signals in real time to maintain and shape the nature of social interactions.

Rodent models of Fetal Alcohol Spectrum Disorder (FASD) show alterations in social behavior [15,16,17,18,19] and communication in the form of USVs [16,17,20,21]. We previously reported that prenatal ethanol exposure decreased play fighting behavior in two separate cohorts of animals [17,22]. In one cohort, adolescent males exposed to ethanol prenatally showed increased latency to emit 50 kHz USVs with no change in the number of USV calls [17]. Thus, the USV data did not fully predict the play behavior, and may suggest that hedonic USVs emitted in a dyad containing an ethanol-exposed rat do not elicit or maintain playful social interactions. This dysregulation was also noted by Spear’s group in adolescent rats following acute exposure to ethanol just prior to social interactions [23]. We also reported that neonatal unilateral whisker clipping (WC) further decreased the number of play fighting interactions by a pair that contained an ethanol-exposed animal [22]. Ethanol-exposed rats appear to be particularly vulnerable to deprivation of sensory input regarding playful social interactions; it is possible that the disrupted relationship between USV social cues and play reflects the inability of these animals to interpret cues from a conspecific and integrate rapidly changing sensory input. This suggests that additional disruption of sensory input, such as WC, would further impair the function of vocalizations and social interaction.

In the current study, we analyzed the USV emissions by these same animals to test the hypothesis that prenatal ethanol exposure disrupts social communication and that communication will be even more disrupted by the combination of prenatal ethanol exposure and WC. Because USVs are emitted from both animals, we also determined play fighting behavior in the previously unreported play partner. Overall, we find that hedonic USVs correlate with the number of playful interactions exhibited within a dyad of typically developing control rats. However, the combination of prenatal ethanol exposure and postnatal WC abolishes this correlation more persistently than either alone.

## 2. Materials and Methods

### 2.1. Subjects

Subjects used in this study are the same as previously described [22]. Dams were timed-pregnant Long Evans rats (Harlan, Frederick, MD, USA), that arrived on gestational day (G)3 (G1 was the day on which a sperm-positive plug was first identified). Animals were housed at the University of Maryland, Baltimore, an Association for Assessment and Accreditation of Laboratory Animal Care International (AAALAC) accredited facility. This facility is humidity (40%–45%) and temperature-controlled (22 °C), and is maintained on a 12/12 h light/dark cycle (lights on at 7 a.m.). All procedures performed were in accordance with the guidelines for animal care established by the National Institutes of Health as well as with the approval of the Institutional Animal Care and Use Committee (IACUC) at the University of Maryland, Baltimore.

### 2.2. Prenatal Exposure

As previously reported [22], on G6 dams were randomly assigned to one of three prenatal exposure conditions. Two groups received a liquid diet (L10251A, Research Diets, NB, Canada); one received diet containing 11.5% ethanol-derived calories (EDC) on G6 and G7 (2.09% v/v ethanol in diet), 22% EDC on G8–G10 (4.27% v/v ethanol in diet), and 35% EDC diet from G11–20 (6.36% v/v ethanol in diet) (ET). Blood ethanol concentrations of dams fed this diet typically reach 100–150 mg/dL [24,25]. The second group was pair fed an isonutritive liquid diet containing maltose in place of ethanol to make it isocaloric (PF). A third group received ad libitum access to laboratory chow (CH). All animals had ad libitum access to water. Liquid diet-fed dams were returned to regular laboratory chow on G21 prior to birth on G22 (also designated postnatal day (P) 0). Dam and litter outcomes are reported elsewhere [22].

### 2.3. Postnatal Manipulations

On P1, all experimental litters were cross-fostered to a surrogate CH-fed dam. At this time litters were culled to 10 maintaining 6:4 male:female sex ratio where possible. One male and one female pup from each litter were randomly assigned to a postnatal unilateral whisker clip (WC) group. The right whiskers were clipped to within 1 mm of the skin on P1, P3, and P5 using sharp surgical scissors. One male and one female pup from each litter served as a non-whisker clipped (NWC) control. On P21, pups were weaned and housed in same sex pairs with their littermates. To avoid potential litter bias, no more than one male and one female from a litter was allotted to each treatment condition per testing age.

### 2.4. Ultrasonic Vocalization Recording

On P28 or P42, experimental pups were weighed and placed in the social interaction test chamber (30 cm × 20 cm × 20 cm; Binghamton Plate Glass, Binghamton, NY, USA). After 30 min an unfamiliar play partner (offspring of a non-manipulated dam, matched for age, sex, and weight ±10 g) was placed in the chamber and the social interaction was recorded for 10 min. USVs were recorded for a subset of animals during the interaction using an ultrasound microphone (Condenser Microphone 116H; Avisoft Bioacoustics, Berlin, Germany). The sampling rate was 250.0 kHz and data were recorded in a 16-bit format. Sonographs were visualized using Avisoft Bioacoustics^™^ sonographic software (Avisoft-RECORDER version 4.2.8 and Avisoft SAS Lab Pro version 5.1; Avisoft Bioacoustics) and were scored by experimenters blinded to condition. The latency to begin vocalizing and the number of 50 kHz calls and 22 kHz calls was determined.

USV outcomes are for the pair of animals involved in the social interaction. To better assess the interaction between play fighting and USVs, we scored play fighting initiated by the untreated play partner and added this score to the outcome for the previously scored experimental animal [22] to generate the total number of play fighting counts for the pair. Thus, we generated play fighting data for the experimental subject, the play partner, and the total for both. Play fighting is defined as tagging (lunging at the other animal with extended forepaws) and pinning (one animal stands over the other that is on its back).

Our previous report [22] showed that the number of play fighting bouts initiated by the experimental animal was lower in both male and female ET animals compared with CH and PF animals at P28 and P42. Play fighting bouts were even lower in ET-WC males than ET-NWC males at both ages, but were only lower in WC females at P28. Recordings of USVs were only available for a subset of animals that underwent the social interaction test. Because this reduced specific group sizes, we combined animals from the chow (CH) and pair fed (PF) groups into a single group called control (CT). We did not find differences between CH and PF groups in prior studies or in statistical comparison of the small subgroups. Play fighting data described in this manuscript are only from the animals used for USV evaluation. Sample sizes are shown in Table 1.

### 2.5. Experimental Design and Data Analysis

For each play fighting measure, a mean and standard error of the mean (SEM) was generated for each group. Play fighting dependent measures were assessed independently using separate between group analyses of variance (ANOVA). Data were separated by age. Thus, the study design was a 2 × 2 × 2 analysis of variance (ANOVA), with the between subjects factors of prenatal exposure (ET or CT), postnatal manipulation (WC or NWC), and sex (male or female). Where the ANOVA identified an overall effect, post hoc comparisons were run using the Tukey test. Follow-up univariate ANOVAs were run on play behavior outcomes to determine the source of the difference.

Correlations were conducted to understand the impact of prenatal ethanol and sensory deprivation on the relationship between expression of 50 kHz USVs and frequency of social play. To do this, Pearson’s *r* was determined using number of 50 kHz and total number of play interactions. Males and females were analyzed together to increase the number of animals in each condition.

Correlations were run using SPSS 22.0 software (IBM Corp., Armonk, NY, USA), all other statistical analysis was done using SigmaPlot 12.3 software (Systat Software Inc., San Jose, CA, USA).

## 3. Results

### 3.1. Play Fighting

Assessment of the total number of play fighting behaviors was determined separately for the experimental subject, the play partner and then combined for both (total play fighting). Data were analyzed using a three-way ANOVA with Prenatal exposure (CT or ET) × Postnatal manipulation (WC or NWC), and Sex as between-subject factors. Because the data for the experimental animals are a subset of the data previously reported on, we analyzed them again and report those outcomes here.

### 3.2. Play Fighting Outcomes at P28

The three-way ANOVA confirmed a significant effect of Prenatal exposure on the number of play fighting behaviors initiated by the experimental animals (F(1,82) = 61.94, *p* < 0.001); animals exposed to ethanol initiated play less often than control animals (*p* < 0.001). A significant effect of Postnatal manipulation was also detected in the number of play fighting behaviors initiated by the experimental subjects (F(1,82) = 18.586, *p* < 0.004). Although the interaction between Prenatal and Postnatal factors failed to reach significance (*p* < 0.066), to determine which groups significantly differed, each group was coded independently and subjected to a univariate ANOVA with Tukey’s post hoc tests. This analysis confirmed that prenatal ethanol exposure significantly (*p* < 0.001) reduced initiation of play compared to controls as did the combination of ethanol and WC (*p* < 0.0001). WC alone did not reduce social play initiation compared to NWC control rats (*p* > 0.05). The combination of prenatal ethanol exposure and WC reduced play initiation significantly (*p* < 0.017) compared to rats subjected to prenatal ethanol alone. Thus, the main effect of WC detected by the three-way ANOVA was driven by the reduction of play initiated by rats subjected to both prenatal ethanol and whisker clipping, rather than being a main effect of WC alone.

Initiation of play by non-exposed play partners showed a significant interaction between Prenatal and Postnatal factors (F(1,82) = 4.251, *p* < 0.042); typically developing rats paired with ET/WC animals initiated significantly less play fighting than other animals (*p* < 0.001).

The total play fighting in each dyad also showed an interaction between Prenatal and Postnatal factors (F(1,82) = 8.802, *p* < 0.004); overall, ET animals played less than CT animals (*p* < 0.007) and ET/WC pairs played less than ET/NWC pairs (*p* < 0.001). There was no significant effect of Sex at this age, and Prenatal × Sex, Prenatal × Sex, and Prenatal × Postnatal × Sex interactions were not significant at this age.

### 3.3. Play Fighting Outcomes at P42

**Figure 1:**
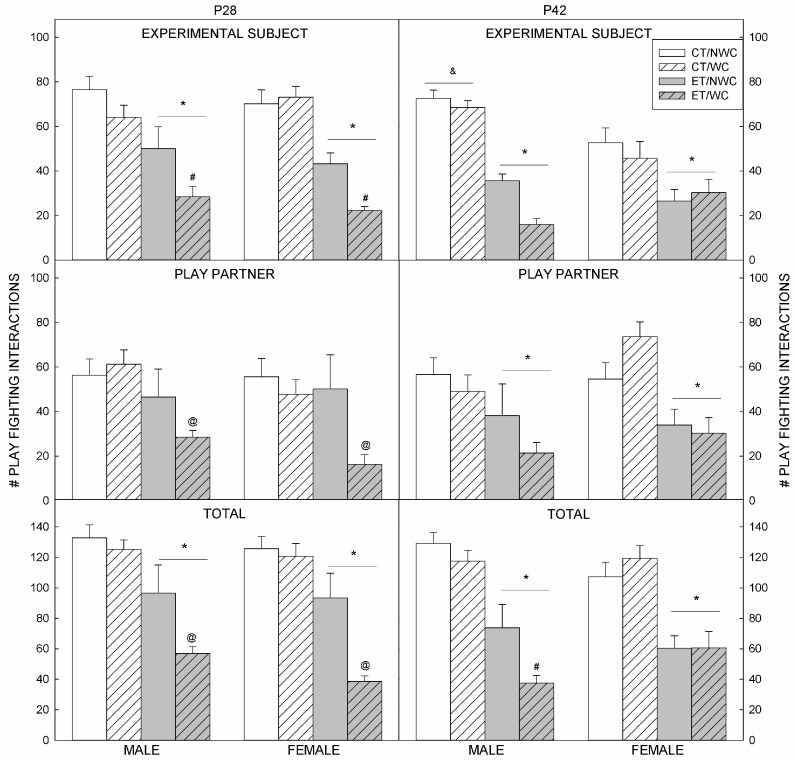
Social interaction. The number of play fighting interactions initiated by the experimental subject (**Top**) or the play partner (**Middle**) during the 10 min social interaction test was counted. The total for both is shown in the bottom graphs. **Left**: At P28, ethanol-exposed animals initiated play fighting less often (**Top**), play partners paired with ET/NWC animals also initiated play fighting less often (**Middle**). Overall, pairs containing ET animals played less than pairs of CT animals, and pairs with an ET/WC rat played less than pairs with an ET/NWC rat. **Right**: On P42, CT males play fought more than CT females (**Top** and **Middle**), ET animals initiated play fighting less frequently than CT animals (**Bottom**). Overall, pairs with an ET animal played less than pairs with CT animals, and WC males played less than NWC males. * Significantly (*p* < 0.05) different to CT animals; # significantly (*p* < 0.05) different to NWC animals; @ significantly (*p* < 0.05) different to same-sex ET/NWC animals. & significantly (*p* < 0.05) different to CT females; CT control, ET ethanol-exposed, NWC non-whisker-clipped, P postnatal day, WC whisker-clipped.

The three-way ANOVA identified a significant Prenatal × Sex interaction in play fighting initiated by the experimental animals (F(1,89) = 6.142, *p* < 0.015); CT males initiated play more than CT females (*p* < 0.053), and ET animals initiated play less than CT animals regardless of sex (*p* < 0.0001) (Figure 1). WC did not affect outcomes in experimental animals at this age.

In play partners, only Prenatal exposure affected the number of play fighting behaviors (F(1,89) = 18.877, *p* < 0.001); CT animals initiated play more than ET animals (*p* < 0.001).

The total play fighting in each dyad showed a significant effect of Prenatal exposure (F(1, 89) = 68.951, *p* < 0.001) and a Postnatal × Sex interaction (F(1,89) = 4.301, *p* < 0.041). Pairs with an ET animal played less than pairs with CT animals (*p* < 0.001), and WC males played less than NWC males (*p* < 0.026).

### 3.4. USV Outcomes at P28

**Figure 2:**
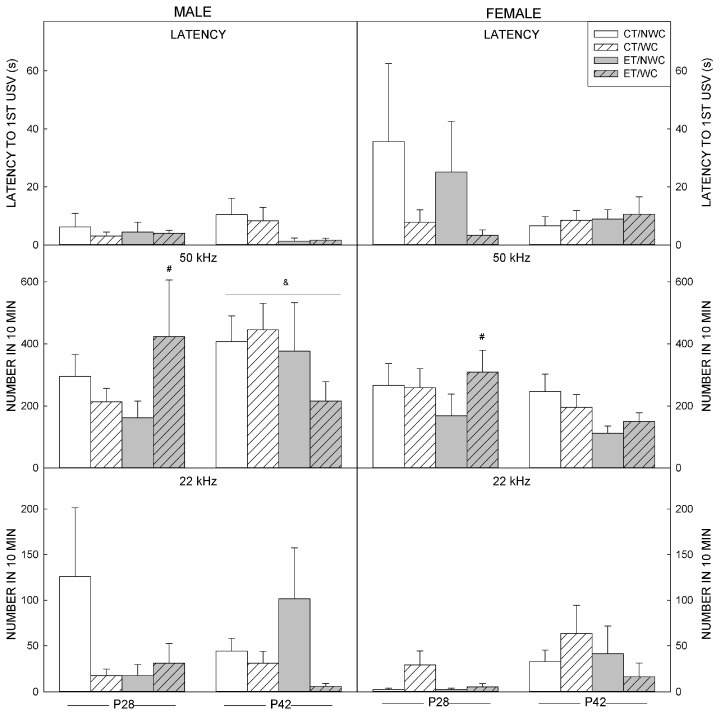
Ultrasonic vocalizations. The latency to first USV (**Top**), and the total number of 50 kHz (**Middle**) and 22 kHz (**Bottom**) USVs were recorded during the 10 min social interaction. **Left**. At P28, there were no differences in the latency to first USV. The number of 50 kHz USVs was higher in ET/WC animals than ET/NWC. The 22 kHz USVs were higher in CT/WC than all other groups in females, but males did not show any between-group differences. **Right**. At P42, there were no effects on latency or 22 kHz USVs. Males showed more 50 kHz calls than females. & significantly (*p* < 0.05) different to females; # significantly (*p* < 0.05) different to NWC animals; CT control, ET ethanol-exposed, NWC non-whisker-clipped, P postnatal day, USV ultrasonic vocalization, WC whisker-clipped.

Latency to the first USV was not significantly different among the groups (Figure 2). The number of 50 kHz USVs showed a Prenatal × Postnatal interaction (F(1,82) = 4.439, *p* < 0.038), where ET/WC animals had more vocalizations than ET/NWC animals (*p* < 0.031). The number of 22 kHz USVs was highly variable, and showed a Prenatal × Postnatal × Sex interaction (F(1,82) = 4.808, *p* < 0.031). This outcome results from a significant Prenatal × Postnatal interaction in females (*p* < 0.001) that was not seen in males (*p* < 0.840). 

### 3.5. USV Outcomes at P42

The three-way ANOVA did not identify a significant effect of any factor on latency to first USV at this age or on the total number of 22 kHz vocalizations (Figure 2). The number of 50 kHz USVs was different between the sexes (F(1,89) = 9.920, *p* < 0.002) with males vocalizing more than females.

### 3.6. Correlations

**Figure 3:**
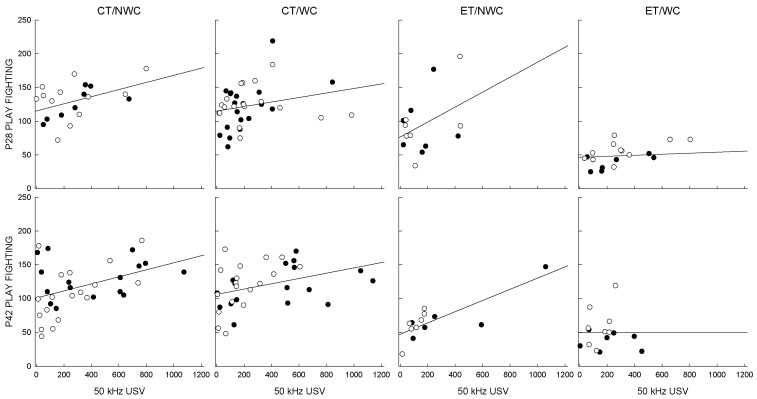
Correlation between play fighting and USVs. The correlation between play fighting and 50 kHz USVs was tested independently for each group. Significant correlations were found for CT/NWC animals at P28, and for CT/NWC, CT/WC, and ET/NWC animals at P42 (Pearson’s *r* and *p*-values are shown in Table 2). Closed circles show male data, open circles show female data.

Assessing the data using Pearson’s *r* coefficient shows that 50 kHz calls significantly and positively correlate with total play fighting (*r* = 0.44, *p* < 0.051) in CT/NWC rats at P28 (Figure 3). This was not evident in WC rats (*p* < 0.15) or in ET animals regardless of whisker clipping (*p* < 0.16). Thus, either prenatal ethanol exposure or postnatal sensory deprivation dysregulated the normal tendency to emit 50 kHz USVs during social play interactions.

The number of 50 kHz USVs significantly correlated with the number of episodes of social play in rats tested at P42 in CT/NWC animals (*p* < 0.014). This relationship was also apparent in CT/WC and ET/NWC rats. However, the combination of prenatal ethanol and postnatal sensory deprivation continued to dysregulate the expression of USVs and social play.

## 4. Discussion

Animals exposed to the combination of prenatal ethanol exposure and neonatal whisker clipping initiated fewer bouts of play fighting during a social interaction test in adolescence and decreased the emission of hedonic USVs. Both males and females showed a suppressive effect of the combined insults on initiation of play at P28. This persisted in males, i.e., was also apparent at P42. A significant correlation between USVs and play behavior was evident in control animals at P28 but this was disrupted by ethanol exposure or by whisker clipping. At P42, the relationship between USV and play fighting appeared to be less labile, and was only disrupted in animals that were exposed to ethanol and whisker clipping.

Social play occurs spontaneously and is inherently rewarding [26,27]. Because play behavior is not necessary for survival, it is generally assumed that it promotes development in a general sense, facilitating cognitive and emotional development, and preparing the organism for social interactions throughout the lifespan [28]. Social isolation and, therefore, deprivation of social play disrupts both cognitive and emotional development (reviewed in [28]). Rats isolated during adolescence and early adulthood perform poorly in tasks in which task demands change and become more challenging [29,30]. Though the long-term effects of reduced intrinsic motivation, relevant to the reduced play induced by prenatal ethanol and sensory deprivation, are less understood, it appears that both forced deprivation through isolation and reduced volition can result in similar patterns of deficits in adulthood. Socially isolated rats and ethanol-exposed rats perform poorly on tasks in which contingencies change, such as reversal learning [31,32,33].

The utility of USVs in social play in adolescent rats remains unresolved. Devocalization of juvenile male rats does not necessarily reduce social play interactions when paired with an intact unfamiliar rat [34]. Thus, reciprocal USVs are not necessary to elicit play. However, playful attacks are more likely to escalate into serious aggressive attacks when one unfamiliar rat of an adult male dyad is devocalized [34]. This supports the notion that hedonic USVs maintain the playfulness of interactions at least between stranger rats [12]. Interestingly, young male adult rats exposed to prenatal ethanol are more aggressive toward male littermates than pairs of control rats [35]. USVs were not measured in that experiment, but our results suggest that this may be due in part to a suppression of this form of communication. Expression of hedonic USVs in adult rats also appears to modulate potential sexual interactions. Females exhibit behaviors indicative of sexual receptivity in response to 50 kHz vocalizations [36]. Similarly, female rats will localize and navigate toward playback of male 50 kHz USVs in a maze, but not toward an amplitude- and time-matched white noise [37]. Expression of USVs during adolescence and early adulthood may serve to prepare rats for these types of interactions in adulthood. Whether adult females are as responsive or receptive to ethanol-exposed males and/or playback of their vocalizations is unknown. Sex behavior in males prenatally exposed to ethanol is not fully masculinized or defeminized, however, suggesting reproductive success is compromised in these animals [38]. Experiments directly assessing the response of females to ethanol-exposed male USVs could potentially clarify the long-term impact of the dysregulation observed here in adolescence.

Long-term sensory deprivation to the somatosensory cortex by whisker clipping produces long-term changes in neuronal responses to whisker stimulation after the whiskers have been allowed to re-grow [39]. Deprived neurons within whisker-specific barrels become hyper-responsive to whisker stimulation, and less selective to whisker orientation [39]. These changes were observed with a much longer period of whisker clipping than used here. However, neonatal whisker clipping similar to that used here can also alter the structure and function of somatosensory cortex: Spiny stellate cells in whisker barrel walls had a larger dendritic span and increased spine density in rats that underwent neonatal whisker clipping compared with control animals [40]. Neonatal whisker clipping also resulted in reduced binding of inhibitory GABAergic drugs and enhanced binding of excitatory glutamatergic drugs in the somatosensory cortex, and this compensatory mechanism persists into adulthood, long after the whiskers have re-grown [41]. Such changes may contribute to the persistent behavioral deficit seen here.

Whisker clipping alone and prenatal ethanol exposure alone disrupted the relationship between expression of USVs and social play in young adolescent animals; only the combination led to a persistent dysregulation of this correlation seen at P42. There are a number of possible reasons for this. To our knowledge, the type of USV that rats can emit does not change between 28 and 42 days of age, but there may be age-related differences in play behavior, the number of USV emissions, and/or the function of the USVs. Hedonic, 50 kHz USVs are emitted in non-aversive situations and may be used to elicit a playful interaction. There was no change in the total number of play fighting interactions at the two ages, but there were more 50 kHz USV emissions by males (CT/NWC, CT/WC and ET/NWC) at P42 compared with P28. This may be related to their peri-pubertal status. It is also possible that there is a difference in USV function during adolescence compared to adulthood.

## 5. Conclusions

In conclusion, animals that were exposed to ethanol prenatally exhibited reduced social play in early and late adolescence but their hedonic vocalizations during play were only reduced in early adolescence. Control rats showed significant correlations between play behavior and hedonic USVs in early and late adolescence. In early adolescence the correlation was disrupted by ethanol-exposure, whisker clipping and the combination. In late adolescence, only the combination abolished the correlation, suggesting that animals may have been able to recover from a single hit but not from both.

## Figures and Tables

**Table 1:** Sample sizes.

-	CT/NWC	CT/WC	ET/NWC	ET/WC
P28 M	8 (3 CH/5 PF)	19 (10 CH/9 PF)	7	8
P28 F	12 (5 CH/7 PF)	18 (10 CH/8 PF)	7	11
P42 M	16 (8 CH/8 PF)	17 (9 CH/8 PF)	6	7
P42 F	18 (9 CH/9 PF)	18 (9 CH/9 PF)	7	8

CT, control (CH from chow-fed dam, PF from pair-fed dam); ET, ethanol; F, female; M, male; NWC, non-whisker-clipped; P, postnatal day; WC, whisker-clipped.

**Table 2:** Number of rats per group and Pearson’s *r* coefficient for each experimental condition.

Age	Condition	*n*	Pearson’s *r*	*p*
P28	CT/NWC	20	0.441	0.051 *
	CT/WC	37	0.240	0.152
	ET/NWC	14	0.396	0.162
	ET/WC	19	0.180	0.461
P42	CT/NWC	34	0.416	0.014 *
	CT/WC	35	0.367	0.030 *
	ET/NWC	13	0.808	0.001 *
	ET/WC	15	0.001	0.998

CT, control animal; ET, ethanol-exposed animal; NWC, non-whisker-clipped; WC, whisker-clipped. * and bold font denote *p* ≤ 0.05.
